# LncRNA CASC11 Promotes Hepatocellular Carcinoma Progression *via* Upregulation of UBE2T in a m^6^A-Dependent Manner

**DOI:** 10.3389/fonc.2021.772671

**Published:** 2021-11-24

**Authors:** Fei Chen, Meijun Li, Liang Wang

**Affiliations:** ^1^ Department of Ultrasound, The First Affiliated Hospital of Jinzhou Medical University, Jinzhou, China; ^2^ Department of Hematology, The Third Affiliated Hospital of Jinzhou Medical University, Jinzhou, China; ^3^ Department of General Surgery, The First Affiliated Hospital of Jinzhou Medical University, Jinzhou, China

**Keywords:** UBE2T, mRNA stability, RNA methylation, posttranscriptional regulation, ALKBH5

## Abstract

Hepatocellular carcinoma (HCC) is one of the most frequent malignancies and the third leading cause of cancer-related deaths worldwide. Besides, it has been revealed that long non-coding RNA (LncRNA) cancer susceptibility candidate 11 (CASC11) is involved in cancer progression. However, the functional role and underlying mechanism of CASC11 in HCC remains largely unknown. In this context, here, it was found that CASC11 was upregulated in HCC tissues and associated with tumor grades, metastasis, and prognosis of HCC patients. Functionally, CASC11 facilitated HCC cell proliferation, migration, and invasion *in vitro*, and enhanced tumor growth and metastasis *in vivo*. Mechanistically, CASC11 associated with and stabilized Ubiquitin-conjugating enzyme E2T (UBE2T) mRNA. To be specific, it decreased UBE2T N^6^-methyladenosine (m^6^A) level *via* recruiting ALKBH5. Moreover, CASC11 inhibited the association between UBE2T mRNA and m^6^A reader protein YTHDF2. Taken together, our findings demonstrate the epigenetic mechanism of CASC11 in the regulation of UBE2T expression and possibly provide a novel therapeutic target for HCC treatment.

## Introduction

Hepatocellular carcinoma (HCC) is one of the most frequent malignancies and the third leading cause of cancer-related deaths worldwide (1). Even though significant progress has been achieved over the past decades in the clinical treatment of HCC, such as surgical resection, transhepatic arterial chemotherapy and embolization (TACE), liver transplantation, targeted therapy, and immunotherapy, the overall survival rate of patients with intermediate- and advanced-stage HCC remains unsatisfactory (2). Thus, it is urgent to understand the underlying mechanism of HCC pathogenesis and progression, and develop novel and effective therapeutic techniques for HCC patients.

Long non-coding RNAs (lncRNAs) are classified as non-coding RNAs that are featured with more than 200 nucleotides in length, without or with limited coding potential. Besides, they have been found to act as important regulators in modulating gene expression *via* multiple mechanisms. For example, lncRNAs associate with histone complexes and regulatory transcriptional proteins, which can remodel the chromatin structure (3, 4), and they also regulate posttranslational modification of their interacting proteins (5). Additionally, lncRNAs may work as competing endogenous RNAs (ceRNAs) or recruit regulatory proteins and interact with mRNAs, then regulating the stability of target mRNA (6). Actually, increasing evidence has shown that lncRNAs are closely related to the occurrence and development of tumors (7). To be specific, abnormal expression of some lncRNAs was found and indicated prognosis in HCC patients, including HEIH, lncRNA-ATB, HOTAIR, HCAL, and lncTCF7 (8–12). Here, it should be noted that LncRNA cancer susceptibility candidate 11 (CASC11) is a novel lncRNA and has been revealed to be upregulated in several human cancers, including colorectal cancer (CRC), gastric cancer, bladder cancer, and ovarian cancer. Apart from that, overexpression of CASC11 also showed poor prognosis of patients. Functionally, CASC11 enhances proliferation and metastasis in cancer cells (13–16). However, the clinical significance, the functional role, and the underlying regulatory mechanism of CASC11 in HCC are still unclear to a great extent.

The current study aims to investigate the expression pattern of CASC11 in HCC tissue samples and the relationship between its expression and the prognosis of HCC patients. Then, it was further revealed that CASC11 enhanced HCC growth and metastasis *via* upregulation of Ubiquitin-conjugating enzyme E2T (UBE2T). In the end, the findings of this study suggest that CASC11 could be a novel therapeutic target for the treatment of HCC.

## Materials and Methods

### Tissue Samples

HCC and the corresponding normal liver tissues were collected from 72 patients who were diagnosed with HCC and underwent curative surgery at the First Affiliated Hospital of Jinzhou Medical University. Besides, the patients were not subjected to other treatment prior to the surgery. Apart from that, informed consent was obtained from all patients, and the study was carried out according to the ethical guidelines of *the Declaration of Helsinki* and was approved by the Ethics Committee of the First Affiliated Hospital of Jinzhou Medical University.

### Cell Culture and Transfection

Human normal hepatocyte THLE-2 cells and HCC cell lines, including Hep3B, Huh7, MHCC97h, SK-Hep-1, PLC/PRF/5, and HCCLM3, were obtained from the Institute of Biochemistry and Cell Biology of the Chinese Academy of Sciences (Shanghai, China). Then, these cells were cultured in DMEM medium (Gibco) with 10% fetal bovine serum (Gibco). Besides, siRNAs were purchased from GenePharma Company (Shanghai, China), and then they were transfected into cells using Lipofectamine™ RNAiMAX Transfection Reagent (Thermo) according to the manufacturer’s instructions. After that, 48 h later, the cells were used for further experiments. As for the target sequences of siRNAs, they were as follows: siALKBH5: GGCTCATCCTTACGTAGTT; siYTHDF2: GCTCTGGATATAGTAGCAATT; siUBE2T: GCTGACATATCCTCAGAATTT.

### Construction of Stable Cells

The lentiviral particles expressing scramble control or CASC11 shRNA (GenePharma, Shanghai, China) were used to knock down CASC11 expression in Hep3B cells, while those expressing the empty vector or CASC11 or mutant CASC11 (GenePharma, Shanghai, China) were infected into Huh7 cells. Apart from that, it should be mentioned that the target sequence of shRNAs was shown as follows: scramble shRNA (shCon): TTCTCCGAACGTGTCACGT; shCASC11-1: TGCAGAAGGTCCGAAGAAA; shCASC11-2: GGTTCAGAGGTGACTATTC. Furthermore, the stable cells were selected using 2 μg/ml puromycin for 2 weeks.

### RNA Extraction and Quantitative Real-Time PCR

The total RNA from tissues or cells was extracted using Trizol reagent (Invitrogen) according to the standard protocol, while the cDNA was synthesized using PrimeScript™ 1st Strand cDNA Synthesis Kit (Takara). In addition, qRT-PCR was carried out on ABI StepOne Plus System (Applied Biosystems) using a standard protocol from the SYBR^®^ Green Premix Pro Taq HS qPCR Kit (Accurate Biology).

### Cell Counting Kit-8 Assay

Firstly, 2.0×10^3^ cells were seeded into a 96-well plate. After that, 10 μl CCK-8 reagent was added into each well at different time points and then incubated for 1 h. Finally, the optical density value was measured at 450 nm using Multiskan™ FC System (Thermo Scientific).

### Transwell Assay

The cell migration and invasion assay was carried out using Transwell chambers (8 μm pore size) without or with Matrigel (BD Bioscience), respectively. To be specific, cells suspending in the 200 μl serum-free DMEM medium were plated into the upper chamber, and DMEM supplemented with 10% FBS was placed into the lower chamber. After 24 h, the cells that passed through the membrane to the lower surface were fixed by 4% paraformaldehyde for half an hour, stained by 0.5% crystal violet for 10 min, and then counted under a microscope (Zeiss).

### RNA Stability Assay

Cells were treated with the RNA synthesis inhibitor, Actinomycin D (2 μg/ml). At different time points, the RNA from cells was extracted, and then the loss of UBE2T mRNA was detected by qRT-PCR.

### Western Blot

Proteins were extracted by a RIPA lysis buffer (Beyotime Company) and quantified by a bicinchoninic acid (BCA) protein quantification kit (Thermo). Besides, protein lysates were separated using SDS-PAGE and transferred onto a PVDF membrane (Millipore) that then was blocked with 5% non-fat milk and incubated with specific primary antibodies overnight at 4°C. After washing, the membrane was incubated with their corresponding second antibodies (Jackson), followed by the bands being visualized by the Immobilon Western Chemilum HRP Substrate (Millipore). As for anti-UBE2T, anti-ALKBH5, anti-FTO, anti-YTHDF2, and anti-GAPDH antibodies, they were used and purchased from Abcam.

### Luciferase Reporter Assay

The full-length transcript of UBE2T was cloned and inserted into pmirGLO plasmid (Promega). Apart from that, the mutant UBE2T reporter plasmid was constructed by replacing the adenosine bases within the m^6^A consensus sequences to cytosine. Then, wild-type and mutant UBE2T reporter plasmid was transfected into cells. After 48 h, cells were assayed with Dual-Luciferase™ Reporter (DLR™) Assay Systems. Furthermore, luciferase activity was measured and calculated as the relative ratio of firefly luciferase activity to renilla luciferase activity.

### RNA Immunoprecipitation

For detection of the interaction between CASC11 and UBE2T mRNA, MS2bs (MS2-binding protein)-MS2bp (MS2-binding sequences)-based RIP was performed according to the previous studies (9, 17). Detailedly, cells were transfected with pLV-MS2, pLV-CASC11-MS2, or pLV-CASC11-mut-MS2, and pMS2-GFP (Addgene). After 48 h, cells were used to perform the RIP assay *via* a GFP antibody (Roach) and the Magna RIP RNA-Binding Protein Immunoprecipitation Kit (Millipore) according to the manufacturer’s instruction. Besides, the RNA pulled down by CASC11 was detected using qRT-PCR or employed for RNA sequencing (BGI Group, Guangdong, China). For identifying the interaction of CASC11 or UBE2T mRNA with ALKBH5, FTO or YTHDF2, cells were used to conduct RIP under the help of the Magna RIP RNA-Binding Protein Immunoprecipitation Kit (Millipore) according to the manufacturer’s instructions. Here, it should be mentioned that anti-ALKBH5, anti-FTO, anti-YTHDF2, and negative control IgG antibodies were purchased from Abcam.

### RNA Pull-Down Assay

The RNA pull-down assay was performed as previously described (9). In brief, wild-type or mutant CASC11 was transcribed *in vitro*, respectively, and biotin-labeled with the Biotin RNA Labeling Mix (Roche) and T7 RNA polymerase (Roche), treated with RNase-free DNase I (Roche), and purified with the RNeasy Mini Kit (Qiagen). Apart from that, 1 mg of whole-cell lysate from cells was incubated with 3 ug of purified biotinylated transcripts for 1 h at 25°C, and complexes were isolated with streptavidin agarose beads (Invitrogen). Moreover, the RNA present in the pull-down material was detected by qRT-PCR analysis.

### Methylated RNA Immunoprecipitation

For measuring the m^6^A modification of UBE2T mRNA, the MeRIP assay was carried out using Magna MeRIP™ m^6^A Kit (Millipore) following the manufacturer’s instructions. The antibody used in this experiment was as follows: m^6^A (Abcam); IgG (Millipore).

### Animal Experiments

Male athymic BALB/c nude mice (4–6 weeks old) were maintained under specific pathogen-free conditions. All animal work was conducted according to national guidelines and approved by the ethical committee of the First Affiliated Hospital of Jinzhou Medical University. For the subcutaneous implantation model, 5×10^6^ control and CASC11 overexpressing Huh7 cells were suspended in the 100 μl serum-free DMEM medium and then injected subcutaneously. Then, the tumor volumes were measured. After 4 weeks, mice were sacrificed and the tumors were weighed. Other than that, for the metastatic model, 1×10^5^ control and CASC11 overexpressing Huh7 cells were suspended in the 50 μl serum-free DMEM medium, followed by being injected into the tail vein of nude mice. After 8 weeks, mice were sacrificed, and the lung tissues were isolated and used for H&E staining. Furthermore, the metastatic nodules in lung tissues were counted under a microscope (Zeiss).

### Statistical Analyses

Statistical analysis was conducted using SPSS 16.0 or Prism, while data were analyzed by the two-tailed student’s t test between two groups or by one-way ANOVA followed by Bonferroni test for multiple comparison. Besides, the relationship between CASC11 expression and prognosis of HCC patients was determined using Kaplan-Meier survival analysis and the log-rank test, whereas the correlation between CASC11 and UBE2T expression was measured using by Pearson correlation analysis, when a p-value less than 0.05 was considered to be statistically significant.

## Results

### Upregulation of CASC11 Correlates With Poor Prognosis of HCC Patients

**Figure 1:**
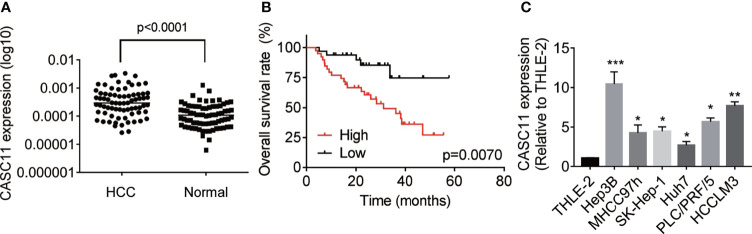
Overexpression of CASC11 indicates poor prognosis in HCC patients. **(A)** qRT-PCR assay was applied to detect the CASC11 expression in 72 pairs of HCC and matched normal liver tissues (Wilcoxon matched-pairs signed rank test). Line indicated the median value of CASC11 expression. **(B)** The patients were divided into CASC11-high and CASC11-low group based on the median value of CASC11 expression in HCC tissues. Kaplan-Meier method and log-rank test was used to evaluate the relationship between CASC11 expression and prognosis of patients with HCC. **(C)** The expression levels of CASC11 between HCC cell lines and human normal hepatocyte THLE-2 cells were tested using qRT-PCR assay. *p < 0.05. Data are mean ± SD. *p < 0.05, **p < 0.01, ***p < 0.001.

To determine the expression pattern of CASC11, firstly, the CASC11 expression in 72 pairs of HCC and matched normal liver tissues was detected. The results showed that HCC tissues exhibited higher CASC11 expression compared with normal liver tissues (**Figure 1A**). Then, the correlation between CASC11 and clinicopathological features of HCC patients was analyzed. To be specific, the patients were divided into CASC11-high and CASC11-low groups based on the median value of CASC11 expression in HCC tissues. As displayed in **Table 1**, CASC11 expression was significantly associated with the tumor grade and metastasis, while Kaplan-Meier method and the log-rank test demonstrated that high CASC11 expression was correlated with the poor overall survival rate of HCC patients to some extent (**Figure 1B**). Furthermore, the expression of CASC11 between HCC cell lines and human normal hepatocyte THLE-2 cells was also tested. Similarly, CASC11 expression was much higher in HCC cell lines than that in THLE-2 cells (**Figure 1C**). Thus, the data suggest that CASC11 may function as an oncogene in HCC.

**Table 1:** The correlation analysis between CASC11 expression and clinicopathological features of HCC patients.

Variables	Category	CASC11 level		P value
		High	Low	
Age	<50	16	12	0.334
	≥50	20	24	
Gender	Male	27	28	0.781
	Female	9	8	
Tumor size	<5 cm	14	15	0.810
	≥5 cm	22	21	
AFP (ng/ml)	<400	5	8	0.358
	≥400	31	28	
HBsAg	Negative	9	10	0.789
	Positive	27	26	
Cirrhosis	Absent	7	9	0.571
	Present	29	27	
Tumor grade	Low	27	17	0.016
	High	9	19	
Metastasis	Yes	21	10	0.009
	No	15	26	

The median of CASC11 level in HCC tissues was used as cutoff.

### CASC11 Promotes HCC Proliferation

**Figure 2:**
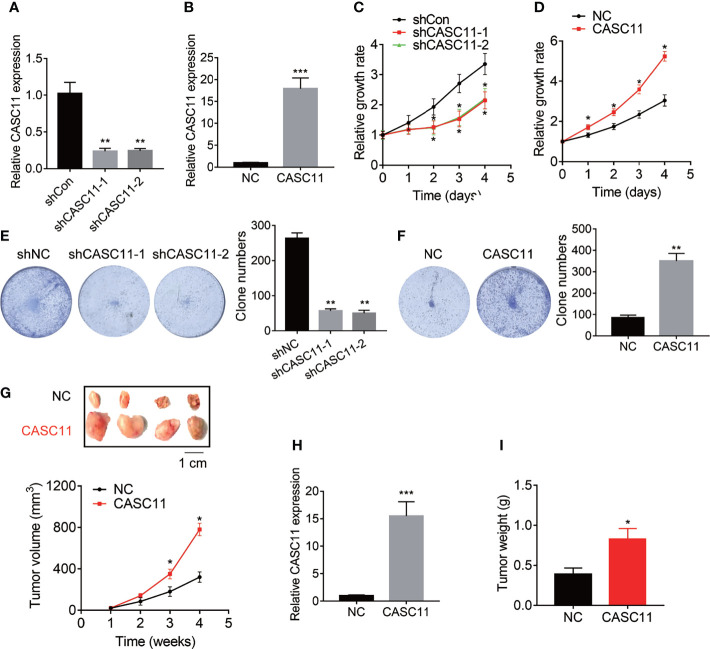
CASC11 promotes HCC cell proliferation *in vitro* and *in vivo*. **(A)** CASC11 was knocked down in Hep3B cells. The knockdown efficiency was validated using qRT-PCR. **(B)** CASC11 was overexpressed in Huh7 cells. The overexpression efficiency was validated using qRT-PCR. **(C)** The CCK8 assay was performed in control and CASC11 knockdown Hep3B cells. **(D)** The CCK8 assay was performed in control and CASC11 overexpressing Huh7 cells. **(E)** The colony formation assay was performed in control and CASC11 knockdown Hep3B cells. **(F)** The colony formation assay was performed in control and CASC11 overexpressing Huh7 cells. **(G)** The tumor growth curve of xenografts formed by control and CASC11 overexpressing Huh7 cells. **(H)** The CASC11 expression in xenografts formed by control and CASC11 overexpressing Huh7 cells was tested using qRT-PCR. **(I)** The tumor weight of xenografts formed by control and CASC11 overexpressing Huh7 cells. Data are mean ± SD. *p < 0.05, **p < 0.01, ***p < 0.001.

The effect of CASC11 on HCC cell proliferation was determined. Among the HCC cell lines (**Figure 1C**), Hep3B cells displayed the highest level of CASC11, while Huh7 cells expressed the lowest CASC11 level. Therefore, Hep3B cells were chosen for knockdown experiments and Huh7 cells were selected for overexpression experiments, respectively. In addition, the knockdown or overexpression efficiency of CASC11 was validated by qRT-PCR (**Figures 2A, B**), whereas the CCK-8 and colony formation assays were performed, and then it was found that knockdown of CASC11 obviously decreased the proliferation rate of Hep3B cells, while overexpression of CASC11 accelerated the Huh7 cell proliferation (**Figures 2C–F**). Furthermore, to confirm the promotion of CASC11 in HCC proliferation *in vivo*, the control and CASC11-overexpressed Huh7 cells were subcutaneously injected into nude mice, and then the tumor growth was examined. The results showed that the Huh7-CASC11 group exhibited larger tumor volume and weight than the control group (**Figures 2G–I**). Taken together, these findings suggest that CASC11 promotes HCC cell proliferation both *in vitro* and *in vivo*.

### CASC11 Enhances HCC Metastasis

**Figure 3:**
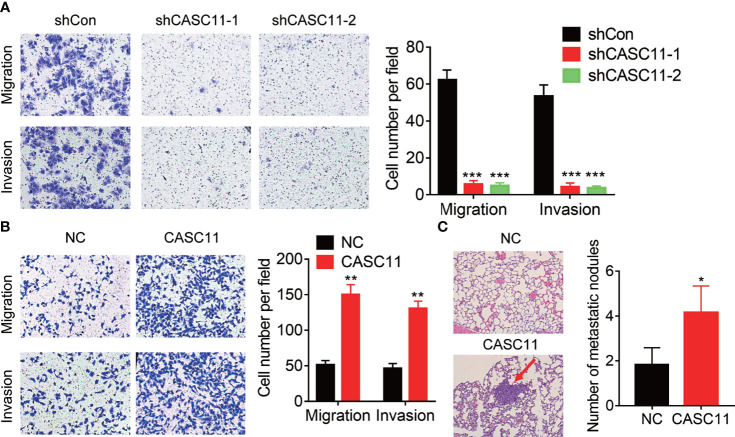
CASC11 promotes HCC metastasis. **(A)** The transwell assay of CASC11 knockdown in Hep3B cells. **(B)** The transwell assay of CASC11 overexpression in Huh7 cells. **(C)** The control and CASC11 overexpressing Huh7 cells were injected into tail vein of nude mice (N=5). After 8 weeks, the pulmonary metastatic nodules were shown and counted. The red arrow indicates the metastatic nodules. Data are mean ± SD. *p < 0.05, **p < 0.01, ***p < 0.001.

The correlation between CASC11 expression and metastasis implied that CASC11 may affect the metastatic ability of HCC cells. To validate this idea, Transwell assays were carried out, and the results demonstrated that knockdown of CASC11 repressed the migration and invasion of Hep3B cells (**Figure 3A**). Conversely, the migratory and invasive capacity was dramatically enhanced by CASC11 overexpression in Huh7 cells (**Figure 3B**). Furthermore, the tail vein injection model was established to prove the pro-metastatic effect of CASC11 *in vivo*. In fact, the CASC11-overexpressed group was featured with more metastatic nodules in lungs than the control group (**Figure 3C**). Collectively, our data reveal a pro-metastatic function of CASC11 in HCC cells *in vitro* and *in vivo*.

### CASC11 Interacts With UBE2T mRNA and Increases Its Stability

**Figure 4:**
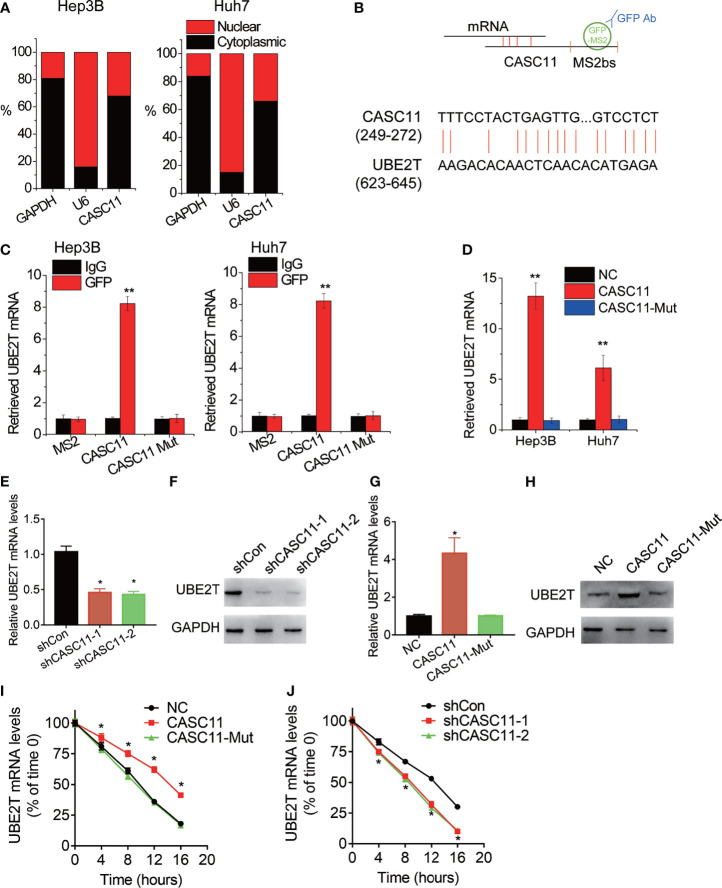
CASC11 associates with and stabilizes UBE2T mRNA. **(A)** The cellular distribution of CASC11 was analyzed. **(B)** The schematic diagram of MS2bs-MS2bp-based RIP (MS2-RIP) experiment (up). The region of putative interaction between CASC11 and UBE2T mRNA was shown (down). **(C)** The UBE2T mRNA associated with CASC11 was detected using MS2-RIP assay. **(D)** Cell lysates were incubated with biotin-labeled CASC11; after pull-down, mRNA was extracted and assessed by qRT-PCR. **(E)** The qRT-PCR analysis of UBE2T mRNA level in control and CASC11 knockdown Hep3B cells. **(F)** The western blot analysis of UBE2T protein level in control and CASC11 knockdown Hep3B cells. **(G)** The qRT-PCR analysis of UBE2T mRNA level in control and CASC11 overexpressing Huh7 cells. **(H)** The western blot analysis of UBE2T protein level in control and CASC11 overexpressing Huh7 cells. **(I)** Control and CASC11 knockdown Hep3B cells were treated with Actinomycin D (5 μg/ml). At different time point, the loss the UBE2T mRNA was detected using qRT-PCR. **(J)** Control and wild-type and mutant CASC11 overexpressing Huh7 cells were treated with Actinomycin D (5 μg/ml). At different time point, the loss the UBE2T mRNA was detected using qRT-PCR. Data are mean ± SD. *p < 0.05, **p < 0.01.

Then, the underlying mechanism of CASC11 regulating HCC growth and metastasis was investigated. Firstly, the cellular distribution of CASC11 in HCC cells was tested, and it was observed that CASC11 was mainly located in the cytoplasm in both Hep3B and Huh7 cells (**Figure 4A**). Besides, the cytoplasm could associate with mRNA, resulting in the stabilization of target mRNA (9). However, whether CASC11 functions in this manner remains unknown. Apart from that, a MS2bs-MS2bp-based RIP assay was also performed to pull down endogenous mRNAs associated with CASC11 (**Figure 4B**), and the retrieved RNA was sequenced. In terms of ubiquitin-conjugating enzyme E2T (UBE2T), it was the most enriched transcript pulled down by CASC11, and has been reported to contribute to tumor growth and metastasis (18). In addition, the highly complementary region between CASC11 and UBE2T mRNA was identified using BLAST (http://blast.ncbi.nlm.nih.gov/) (**Figure 4B**), whereas the binding sites in CASC11 were mutated (named CASC11-Mut). Furthermore, to confirm the direct interaction between CASC11 and UBE2T mRNA, the MS2bs-MS2bp-based RIP assay was performed, and the results demonstrated that the UBE2T mRNA was noticeably enriched by CASC11 in both Huh7 and Hep3B cells than by the empty vector, IgG and CASC11-Mut (**Figure 4C**). Beyond that, the results of the RNA pull-down assay further confirmed the interaction of CASC11 with UBE2T mRNA (**Figure 4D**).

The effect of CASC11 on UBE2T expression was detected by the western blot and qRT-PCR. Then, it was observed that CASC11 knockdown significantly decreased both mRNA and protein level of UBE2T in Hep3B cells (**Figures 4E, F**), while wild-type CASC11, but not mutant CASC11, upregulated UBE2T expression in Huh7 cells (**Figures 4G, H**). To test whether CASC11 regulates the stability of UBE2T mRNA, HCC cells with CASC11 knockdown or overexpression were treated with Actinomycin D to block new RNA synthesis, before the loss of UBE2T mRNA was measured in different time points. After that, it was known that the overexpression of wild-type CASC11 instead of its mutant suppressed the degradation of UBE2T mRNA (**Figure 4I**), whereas silence of CASC11 shortened the half-life of UBE2T mRNA (**Figure 4J**). To sum up, these findings reveal that CASC11 increases UBE2T expression through enhancing the stability of UBE2T mRNA, which depends on the association with UBE2T mRNA.

### CASC11 Regulates m^6^A Modification of UBE2T mRNA *via* Association With RNA Demethylase ALKBH5

**Figure 5:**
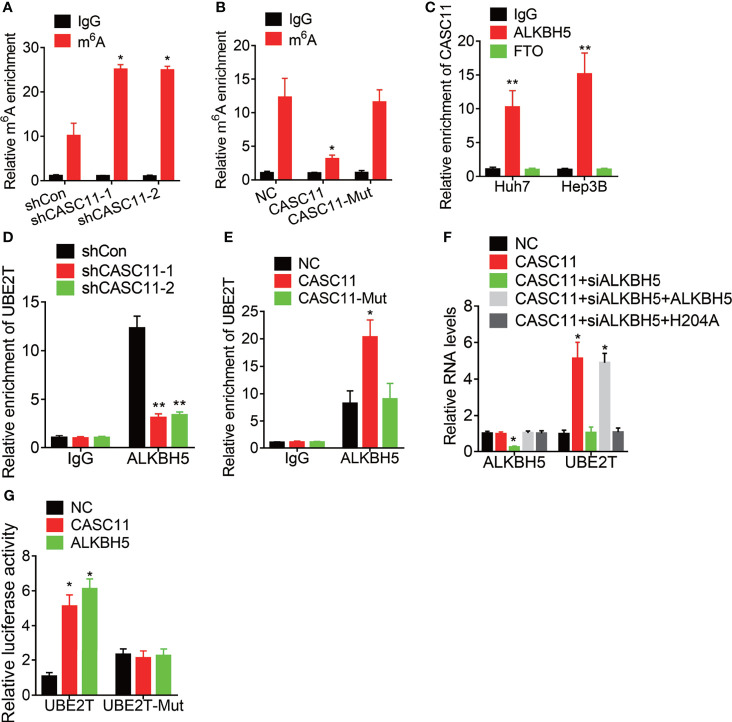
CASC11 decreases the m^6^A modification of UBE2T mRNA *via* association with ALKBH5. **(A)** The m^6^A level of UBE2T mRNA in control and CASC11 knockdown Hep3B cells was examined by MeRIP assay. **(B)** The m^6^A level of UBE2T mRNA in control and wild-type and mutant CASC11 overexpressing Huh7 cells was examined by MeRIP assay. **(C)** The interaction of CASC11 with ALKBH5 or FTO was detected using RIP assay. **(D)** The interaction between ALKBH5 and UBE2T mRNA was tested using RIP assay in control and CASC11 knockdown Hep3B cells. **(E)** The interaction between ALKBH5 and UBE2T mRNA was tested using RIP assay in control and wild-type and mutant CASC11 overexpressing Huh7 cells. **(F)** The mRNA levels of ALKBH5 and UBE2T in indicated Huh7 cells. **(G)** Wild-type or m^6^A consensus sequence mutant UBE2T (UBE2T-Mut) cDNA was constructed and fused with firefly luciferase reporter pmirGLO. Mutation of m^6^A consensus sequences or overexpression of CASC11 or ALKBH5 enhanced the luciferase activity of UBE2T reporter in Huh7 cells. Data are mean ± SD. *p < 0.05, **p < 0.01.

Then, the mechanism of CASC11-mediated stability of UBE2T mRNA was explored. Increasing evidence has demonstrated that N^6^-methyladenosine (m^6^A) modification is involved in the mRNA decay process (19, 20). To testify whether CASC11 modulates UBE2T mRNA stability through this manner, the MeRIP assay was performed. Then, it was found that depletion of CASC11 expression increased the m^6^A level of UBE2T mRNA (**Figure 5A**) to a great degree, while overexpression of wild-type CASC11, instead of mutant CASC11, obviously decreased m^6^A modification of UBE2T mRNA (**Figure 5B**), implying that CASC11 might recruit RNA demethylase to UBE2T mRNA when the m^6^A demethylases include FTO and ALKBH5. In this case, to validate which demethylase was involved in this process, RIP assays using FTO or ALKBH5 antibody were carried out. After that, it was discovered that CASC11 could only be observably enriched by ALKBH5 antibody in both Huh7 and Hep3B cells (**Figure 5C**). Moreover, CASC11 knockdown repressed the association between ALKBH5 and UBE2T mRNA (**Figure 5D**), while the interaction of them was enhanced by upregulation of wild-type CASC11, but not mutant CASC11 (**Figure 5E**). Additionally, siRNA targeting ALKBH5 3’UTR (siALKBH5) was transfected into CASC11-overexpressed Huh7 cells, whereas the CASC11-mediated upregulation of UBE2T was attenuated by ALKBH5 siRNAs. Besides, restoration of ALKBH5 expression rather than catalytic-inactive mutant ALKBH5 (H204A) reversed this effect (**Figure 5F**).

To further address the m^6^A modification of UBE2T mRNA, both wild-type and mutant UBE2T reporters were constructed. Then, to construct the mutant form of UBE2T, the adenosine bases in m^6^A consensus sequences (i.e., RRACH) were replaced by cytosine, which abolished m^6^A modification. Apart from that, the wild-type or mutant UBE2T-fused reporter was transfected into Huh7 cells. When compared to the wild-type, mutation on the m^6^A consensus sequences increased the luciferase activity of UBE2T. Moreover, luciferase activity of the wild-type UBE2T-fused reporter was significantly increased after CASC11 overexpression or ALKBH5. However, upregulation of CASC11 or ALKBH5 did not affect the luciferase activity of the mutant UBE2T-fused reporter (**Figure 5G**). These results prove that CASC11 strengthens UBE2T mRNA stability *via* ALKBH5-mediated m^6^A demethylation.

### CASC11 Attenuates the Association Between YTHDF2 and UBE2T mRNA

**Figure 6:**
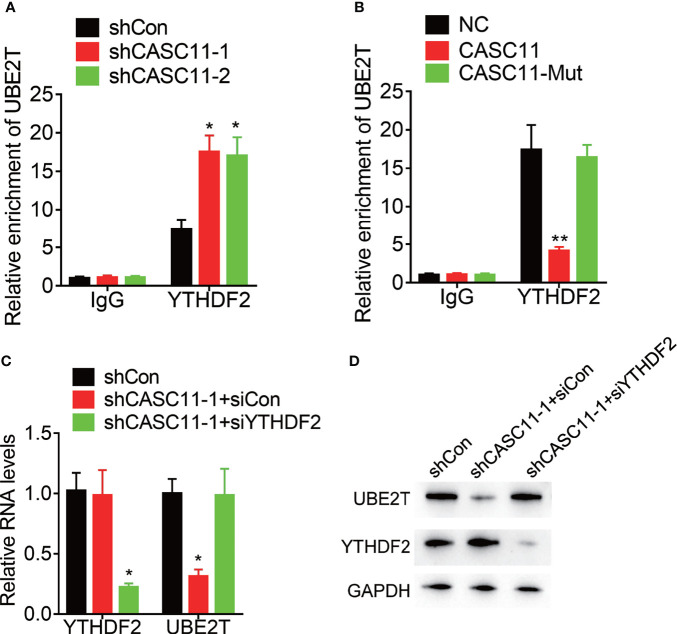
CASC11 attenuates the association between YTHDF2 and UBE2T mRNA. **(A)** The interaction between YTHDF2 and UBE2T mRNA was detected in control and CASC11 knockdown Hep3B cells using RIP assay. **(B)** The interaction between YTHDF2 and UBE2T mRNA was detected in control and wild-type and mutant CASC11 overexpressing Huh7 cells using RIP assay. **(C)** The CASC11 knockdown Hep3B cells were transfected with YTHDF2 siRNA, and the mRNA level of UBE2T was detected using qRT-PCR. **(D)** The CASC11 knockdown Hep3B cells were transfected with YTHDF2 siRNA, and the protein level of UBE2T was detected using qRT-PCR. Data are mean ± SD. *p < 0.05, **p < 0.01.

YTH N^6^-methyladenosine RNA binding protein 2 (YTHDF2), the m^6^A reader protein, selectively recognizes and binds m^6^A-containing mRNA for the purpose of promoting mRNA degradation. Then, the effect of CASC11 on the interaction between YTHDF2 and UBE2T mRNA was confirmed. YTHDF2 could associate with UBE2T mRNA, which was enhanced by CASC11 knockdown in Hep3B cells, but suppressed by CASC11 overexpression in Huh7 cells (**Figures 6A, B**). Furthermore, siRNAs targeting YTHDF2 were transfected into Hep3B cells with CASC11 knockdown. Besides, depletion of YTHDF2 expression upregulated UBE2T expression, which was inhibited by CASC11 knockdown (**Figures 6C, D**). Together, our findings show that CASC11-mediated m^6^A demethylation enhances UBE2T expression through suppressing the YTHDF2-dependent mRNA degradation.

### CASC11 Promotes Malignant Phenotypes of HCC Cells *via* UBE2T

**Figure 7:**
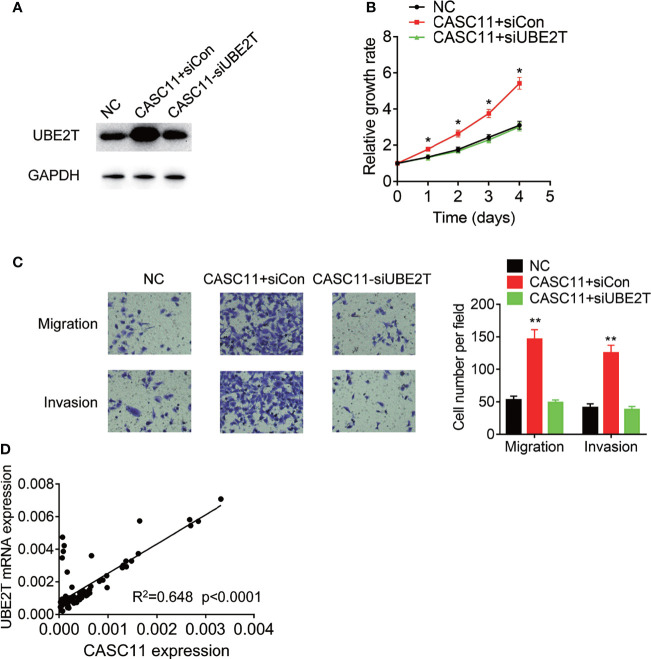
CASC11 promotes malignant phenotypes of HCC cells *via* UBE2T. **(A)** The CASC11 overexpressing Huh7 cells were transfected with UBE2T siRNA. The protein level of UBE2T was detected using western blot. **(B)** The CASC11 overexpressing Huh7 cells were transfected with UBE2T siRNA. The cell proliferation was detected using CCK-8 assay. **(C)** The CASC11 overexpressing Huh7 cells were transfected with UBE2T siRNA. The cell migration and invasion was detected using transwell assay. **(D)** The correlation analysis of CASC11 and UBE2T mRNA in 72 HCC tissues. Data are mean ± SD. *p < 0.05, **p < 0.01.

Finally, rescue experiments were carried out to examine whether CASC11 promotes malignant phenotypes of HCC cells through UBE2T. Knockdown of UBE2T significantly abolished the proliferation, migration, and invasion, which was enhanced by CASC11 overexpression in Huh7 cells (**Figures 7A–C**). Besides, the pathological association between CASC11 and UBE2T was also examined in 72 HCC tissues, and after that, the statistical analysis revealed a positive correlation of CASC11 with UBE2T mRNA expression (**Figure 7D**), supporting that CASC11 functions as an oncogene *via* regulation of UBE2T in HCC.

## Discussion

With the development of high-throughput sequencing technology, the relationship between lncRNAs and tumorigenesis has attracted increasing attention. CASC11 acts as an oncogene in several kinds of human cancers. Here, our report demonstrated that CASC11 was upregulated in HCC tissues and correlated with the tumor grade and metastasis. In addition, elevated expression of CASC11 indicated poor prognosis of patients with HCC. Similarly, CASC11 knockdown decreased HCC cell proliferation, migration and invasion *in vitro*, while ectopic expression of CASC11 exerted the opposite effects. Moreover, CASC11 overexpression facilitated xenograft tumor growth and metastasis *in vivo*.

Recently, the molecular mechanism of CASC11 has been well elucidated in some cancers. In CRC, CASC11 interacts with hnRNP-K and activates the WNT/β-catenin pathway, thus facilitating growth and metastasis (15). Besides, it promotes bladder cancer cell proliferation *via* sponging miR-150 (14), and also mediates the upregulation of TGF-β1, causing the increasing stemness of small-cell lung cancer (21). In HCC, CASC11 has been found to associate with EZH2, thus suppressing the expression of tumor suppressor PTEN (22). Specifically, it functions as a ceRNA of miR-21 and miR-188-5p (23, 24). Moreover, CASC11 recruits EIF4A3 to enhance the stability of E2F1 mRNA (25). In the present study, a novel mechanism of CASC11 in HCC was revealed. The cellular localization of lncRNAs is important for their function. Here, it was found that CASC11 was mainly located in cytoplasm of HCC cells, where LncRNAs typically participate in post-transcriptional regulation by associating with microRNAs or mRNAs (9, 26). Furthermore, our data showed that CASC11 was physically related to UBE2T mRNA, which increased UBE2T expression *via* suppressing the degradation of UBE2T mRNA. The positive correlation between CASC11 and UBE2T mRNA in HCC tissues further supported that UBE2T was a downstream target of CASC11.

To date, over 100 types of chemical modifications have been identified in RNAs. Of them, as the most abundant internal modification on mRNAs, m^6^A is a reversible and dynamic RNA modification, and plays a critical role in virtually all major normal bioprocesses (27, 28). Besides, emerging evidence has been reported in revealing the importance of dysregulated m^6^A modification in cancer initiation and progression. For instance, METTL14, a component of m^6^A methyltransferase complex, plays a tumor-suppressor role in HCC, in which METTL14 and m^6^A levels were decreased in HCC tissues. In addition to that, it suppresses HCC metastasis through m^6^A-dependent modulation of primary miR-126 processing by interaction with DGCR8 (29). Under non-hypoxic conditions, ALKBH5 promotes mRNA stability and expression of NANOG by catalyzing m^6^A demethylation, thus leading to the enrichment of breast cancer stem cells (30). Moreover, our current study revealed that CASC11 recruited ALKBH5 to UBE2T mRNA, reducing the m^6^A level of UBE2T mRNA and then suppressing its binding with YTHDF2. Apart from that, the similar mechanism was also reported by previous studies (31, 32), combiningly demonstrating an important role of lncRNAs in regulating m^6^A modification of mRNAs.

UBE2T acts as an oncogene and is observed to be upregulated in multiple types of cancers (33–37). Besides, it is involved in regulating proliferation, cell cycle modulation, epithelial-mesenchymal transition and invasion (18, 38, 39). As a member of the E2 family, UBE2T participates in the conjugating ubiquitin process and modulates several physiological and pathological processes in a E2-enzyme dependent manner. For example, UBE2T increased H2AX monoubiquitination upon ionizing radiation exposure, and then maintained CHK1 activation and promoted G2/M arrest, thus resulting in increased DNA damage response and HCC radioresistance (40). Other than that, UBE2T accelerates proliferation *via* polyubiquitinating and degrading BRCA1 in breast cancer cells (33), while it promotes DNA crosslinking-induced damage repair *via* monoubiquitinating FANCD2 (41, 42). However, people have still known little about the regulatory of UBE2T expression. Chen et al. reported that circular RNA circ_0090049 acts a ceRNA of miR-605-5p or miR-548c-3p to regulate UBE2T expression (43). E2F5 can activate the UBE2T transcription (44). Additionally, miR-1305 targets UBE2T mRNA to inhibit its expression (45). Whereas, whether the RNA modification is involved in regulating UBE2T expression remains largely unknown. Considering that, our research is the first to reveal that CASC11 regulated UBE2T expression in a m^6^A-dependent manner, and CASC11 promoted proliferation and metastasis *via* UBE2T.

## Conclusion

**Figure 8:**
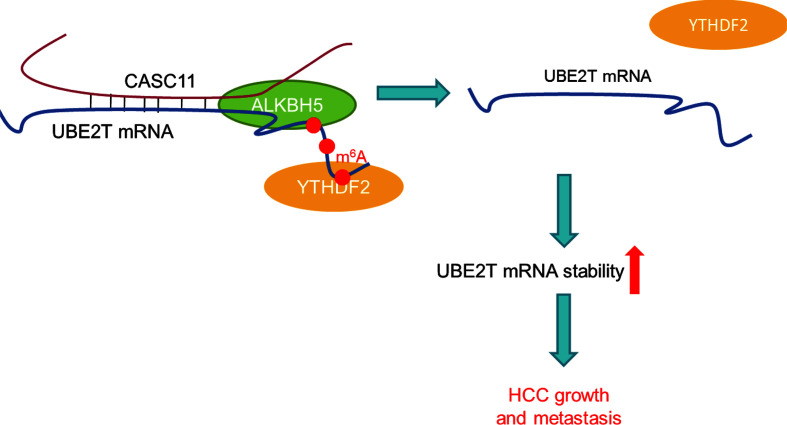
The schematic model of CASC11 promotes HCC progression and its functional mechanism.

In summary, compelling evidences are provided for proving that CASC11 could regulate UBE2T mRNA stability *via* ALKBH5-mediated m^6^A demethylation (**Figure 8**). Besides, the findings of this study have significant implications regarding the role of lncRNAs-mediated m^6^A modification in modulating HCC progression, and also indicate that the CASC11-ALKBH5-UBE2T axis may be a novel therapeutic target for HCC treatment.

## Data Availability Statement

The original contributions presented in the study are included in the article/supplementary material. Further inquiries can be directed to the corresponding author.

## Ethics Statement

The studies involving human participants were reviewed and approved by the Ethics Committee of the First Affiliated Hospital of Jinzhou Medical University. The patients/participants provided their written informed consent to participate in this study. The animal study was reviewed and approved by the ethical committee of the First Affiliated Hospital of Jinzhou Medical University.

## Author Contributions

FC and LW designed the research. FC, ML, and LW performed experiments and analyzed the data. LW wrote the main manuscript. All authors contributed to the article and approved the submitted version.

## Funding

This research was supported by National Natural Science Foundation of China (No. 81902452) and Natural Science Foundation of Liaoning Province (No. 2019-ZD-0803 and 2020-MS-276).

## Conflict of Interest

The authors declare that the research was conducted in the absence of any commercial or financial relationships that could be construed as a potential conflict of interest.

## Publisher’s Note

All claims expressed in this article are solely those of the authors and do not necessarily represent those of their affiliated organizations, or those of the publisher, the editors and the reviewers. Any product that may be evaluated in this article, or claim that may be made by its manufacturer, is not guaranteed or endorsed by the publisher.

## Abbreviations

HCC, hepatocellular carcinoma; CASC11, Long non-coding RNA cancer susceptibility candidate 11; CCK-8, Cell Counting Kit-8; TACE, transhepatic arterial chemotherapy and embolization; lncRNAs, long non-coding RNAs; qRT-PCR, quantitative real-time PCR; UBE2T, ubiquitin-conjugating enzyme E2T; CRC, colorectal cancer.
